# Use of Video in Telephone Triage in Out-of-Hours Primary Care: Register-Based Study

**DOI:** 10.2196/47039

**Published:** 2024-04-04

**Authors:** Mette Amalie Nebsbjerg, Claus Høstrup Vestergaard, Katrine Bjørnshave Bomholt, Morten Bondo Christensen, Linda Huibers

**Affiliations:** 1 Research Unit for General Practice, Aarhus, Denmark; 2 Department of Public Health, Aarhus University, Aarhus C, Denmark

**Keywords:** primary health care, after-hours care, referral and consultation, general practitioner, GP, triage, remote consultation, telemedicine

## Abstract

**Background:**

Out-of-hours primary care (OOH-PC) is challenging due to high workloads, workforce shortages, and long waiting and transportation times for patients. Use of video enables triage professionals to visually assess patients, potentially ending more contacts in a telephone triage contact instead of referring patients to more resource-demanding clinic consultations or home visits. Thus, video use may help reduce use of health care resources in OOH-PC.

**Objective:**

This study aimed to investigate video use in telephone triage contacts to OOH-PC in Denmark by studying rate of use and potential associations between video use and patient- and contact-related characteristics and between video use and triage outcomes and follow-up contacts. We hypothesized that video use could serve to reduce use of health care resources in OOH-PC.

**Methods:**

This register-based study included all telephone triage contacts to OOH-PC in 4 of the 5 Danish regions from March 15, 2020, to December 1, 2021. We linked data from the OOH-PC electronic registration systems to national registers and identified telephone triage contacts with video use (video contact) and without video use (telephone contact). Calculating crude incidence rate ratios and adjusted incidence rate ratios (aIRRs), we investigated the association between patient- and contact-related characteristics and video contacts and measured the frequency of different triage outcomes and follow-up contacts after video contact compared to telephone contact.

**Results:**

Of 2,900,566 identified telephone triage contacts to OOH-PC, 9.5% (n=275,203) were conducted as video contacts. The frequency of video contact was unevenly distributed across patient- and contact-related characteristics; it was used more often for employed young patients without comorbidities who contacted OOH-PC more than 4 hours before the opening hours of daytime general practice. Compared to telephone contacts, notably more video contacts ended with advice and self-care (aIRR 1.21, 95% CI 1.21-1.21) and no follow-up contact (aIRR 1.08, 95% CI 1.08-1.09).

**Conclusions:**

This study supports our hypothesis that video contacts could reduce use of health care resources in OOH-PC. Video use lowered the frequency of referrals to a clinic consultation or a home visit and also lowered the frequency of follow-up contacts. However, the results could be biased due to confounding by indication, reflecting that triage GPs use video for a specific set of reasons for encounters.

## Introduction

General practice serves as a gatekeeper to secondary care in many countries [1]. However, the services in out-of-hours primary care (OOH-PC) are challenging due to high workloads, workforce shortages, and long waiting and transportation times for patients. This development has received much political attention and has caused public debate and reorganization [2,3].

Existing health care systems are currently undergoing a digital transformation, which was pushed by the COVID-19 pandemic [4-9]. As a central part of this digitization, video consultations have been implemented broadly in general practice [5,8-12]. Many countries have introduced video as part of telephone triage in OOH-PC [12-14]. Video use enables triage professionals to visually assess patients, which may imply that more contacts can be ended in a telephone triage contact instead of referring patients to clinic consultations or home visits, which demand more resources. Thereby, video use might reduce use of health care resources related to clinic consultations and home visits.

Research has shown that patients welcome the use of video in general practice in the daytime and also after hours [4,14-16]. However, in daytime general practice, general practitioners (GPs) experience both benefits of (eg, care delivery) and barriers to (eg, technical difficulties, varying suitability for different health problems and patient groups) video use [6,10,16-19]. Two qualitative studies indicated that video use in OOH-PC is beneficial to both triage professionals (eg, it improved patient assessment and reassurance) [13,14] and patients (eg, it led to better reassurance and higher satisfaction) [14]. Two register-based studies found that video use in OOH-PC increased during the COVID-19 pandemic [12,20]. However, little is still known about video use and its effects. This study aimed to investigate video use in telephone triage contacts to OOH-PC in Denmark by studying rate of use and potential associations between video use and patient- and contact-related characteristics and between video use and triage outcomes and follow-up contacts.

## Methods

### Design and Population

We conducted a register-based study of video use in telephone triage contacts to OOH-PC in 4 of the 5 Danish regions (North Denmark Region, Central Denmark Region, Region of Southern Denmark, and Region Zealand). As the Capital Region of Denmark runs a different OOH-PC system than the other 4 regions, this region was not included in this study. We included all telephone contacts from March 15, 2020, to December 1, 2021, and followed each patient for 7 days to record the outcomes. In Region Zealand, telephone contacts were included from March 1, 2021, because this region started using video from this date.

### Setting

Denmark has free public health care for its residents. The health care system is centrally regulated, but most services are provided by the local governments of the 5 regions. Outside office hours, Danish GPs and GP trainees cover shifts in the regional OOH-PC service, which is open on weekdays from 4 PM to 8 AM and 24 hours during weekends and holidays. GPs and GP trainees in their last year of specialist training (hereinafter referred to jointly as triage GPs) perform telephone triage and determine the triage outcome: telephone triage with video use (video contacts) or telephone triage without video use (telephone contacts), clinic consultation, home visit, or hospital admission. The triage GPs assesses whether the problem is suitable for a video contact. If so and if the patient approves, a video link is sent to the patient via text message. When the link is activated, the triage GP can see the patient, but the patient cannot see the triage GP. Triage GPs are paid a fee for service using remuneration codes.

### Outcome Measures

The following outcome measures were defined: the proportion of video contacts (number of video contacts per 100 telephone contacts); the association between video contact and patient- and contact-related characteristics (sex and age of the patient, cohabitation status, comorbidity, educational level, ethnicity, income, urbanization, employment status, region, and time of contact); the frequency of triage outcomes (advice and self-care, referral to clinic consultation, home visit, or hospital admission) and their association with video contact; and the frequency of follow-up contacts in daytime general practice or OOH-PC within 7 days or a hospital admission within 1 day and their association with video contact.

### Data Collection

We used data from the OOH-PC electronic registration system, which provided information on date, time, region, type of contact (telephone contact or video contact), and triage outcome (advice and self-care, referral to clinic consultation, home visit, or hospital admission). We constructed a “time of contact” variable, which was defined by its relation to the next opening time of daytime general practice and dichotomized into >4 hours or ≤4 hours, as the option to refer a patient to their regular GP may influence the triage decision.

To investigate follow-up contacts, we linked data from the OOH-PC registration system to 2 Danish national registers using each patient’s unique personal identification number [21]. The Danish National Health Service Register [22] provided information on date and type of contact to daytime general practice (telephone contacts, video contacts, clinic consultations, or home visits). The Danish National Patient Registry [23] provided information on date of contact to the hospital (emergency department visits and unscheduled hospital admissions) and comorbidity. Comorbidity was defined as the number of diagnoses from the Charlson Comorbidity Index that were recorded as diagnosis codes in hospital charts. Data on socioeconomic characteristics of the patients (sex, age, cohabitation status, educational level, ethnicity, income, urbanization, and employment status) were obtained from Statistics Denmark [24]. All covariates (except for age, sex, and comorbidity) were reported at the household level. For example, household educational level was determined by the member with the longest education. Hence, it was possible to avoid excluding contacts involving children because of missing values. We included only persons with registered socioeconomic characteristics.

### Data Analyses

People with more than 25 contacts to OOH-PC during the study period (comprising 98,126/2,900,566 contacts, 3.4%) were excluded from the data analyses since they were considered outliers. Likewise, people aged >104 years (162/2,900,566 contacts, 0%) and patients with missing covariates (18,740/2,900,566 contacts, 0.7%) were excluded.

**Figure 1.**
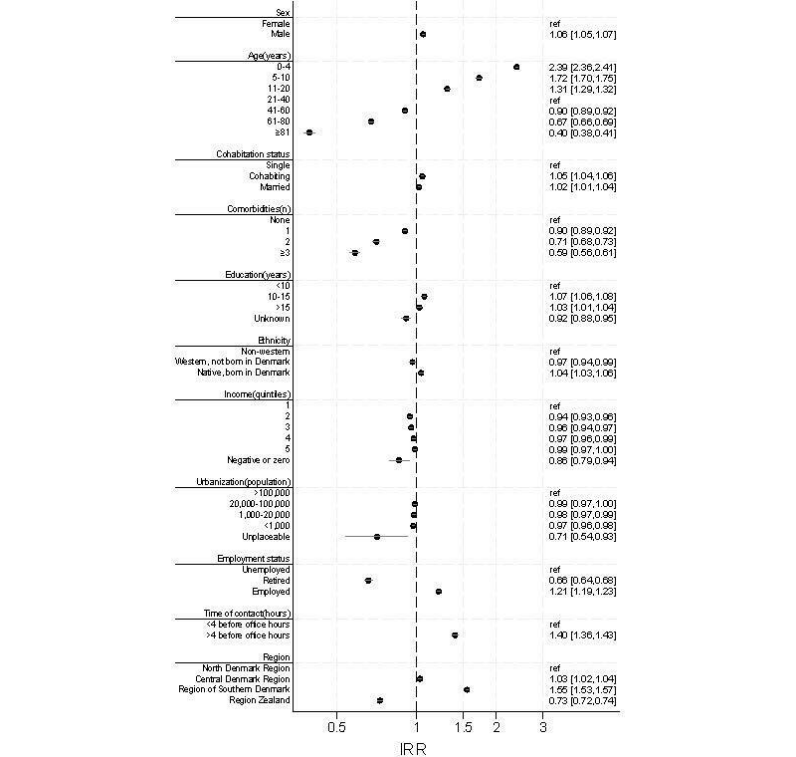
Forest plot presenting the association between patient- and contact-related characteristics and the likelihood of having a video contact (incidence rate ratios [IRRs] with 95% CIs). An IRR >1 indicates a higher use of video contacts compared to the reference group, marked in the right column of the figure. Conversely, an IRR <1 indicates a lower use of video contacts.

Descriptive analyses were used to describe the study population. To ensure convergence of the regressions, we used Poisson regression models to measure the association between patient- and contact-related characteristics and video contacts, and we calculated incidence rate ratios (IRRs) and 95% CIs [25]. Results are presented as a forest plot (Figure 1). Using a Poisson regression model, we also calculated crude and adjusted IRRs (aIRRs) of triage outcomes and follow-up contacts after a video contact compared to after a telephone contact. IRRs were adjusted for patient- and contact-related characteristics. Stata (version 17; StataCorp) was used to analyze all data. Reporting of results was conducted in accordance with the STROBE (Strengthening the Reporting of Observational Studies in Epidemiology) statement.

### Ethical Considerations

The Committee on Health Research Ethics in the Central Denmark Region approved the data collection from the electronic patient records in the OOH-PC registration system (1-45-70-22-22) without informed consent from participants or any provision for them to opt out. The study was listed in the record of processing activities at the Research Unit for General Practice in Aarhus in accordance with the provisions of the General Data Protection Regulation (GDPR). All data on participants were deidentified. Finally, conduct of the study was endorsed by the regional association of GPs.

## Results

### Study Population

During the study period, 2,900,566 telephone triage contacts to OOH-PC were identified (Table 1). Patient- and contact-related characteristics varied between telephone and video contacts; the largest variation was seen for patient age, comorbidity, employment status, region, and time of contact.

**Table 1.** Distribution of patient- and contact-related characteristics (N=2,900,566).

Characteristics		Telephone contacts (n=2,625,363, 90.5%), n (%)	Video contacts (n=275,203, 9.5%), n (%)	Total (n=2,900,566, 100%), n (%)
**Sex**				
	Female	1,427,918 (54.4)	140,684 (51.1)	1,568,602 (54.1)
	Male	1,197,445 (45.6)	134,519 (48.9)	1,331,964 (45.9)
**Age (years)**				
	0-4	311,749 (11.9)	83,672 (30.4)	395,421 (13.6)
	5-10	138,745 (5.3)	25,196 (9.2)	163,941 (5.7)
	11-20	310,179 (11.8)	40,830 (14.8)	351,009 (12.1)
	21-40	688,182 (26.2)	64,780 (23.5)	752,962 (26)
	41-60	508,558 (19.4)	40,955 (14.9)	549,513 (18.9)
	61-80	433,400 (16.5)	16,042 (5.8)	449,442 (15.5)
	≥81	234,550 (8.9)	3728 (1.4)	238,278 (8.2)
**Cohabitation status**				
	Single	942,003 (35.9)	70,266 (25.5)	1,012,269 (34.9)
	Cohabiting	488,499 (18.6)	69,928 (25.4)	558,427 (19.3)
	Married	1,194,861 (45.5)	135,009 (49.1)	1,329,870 (45.8)
**Comorbidities (n)**				
	None	1,946,569 (74.1)	241,173 (87.6)	2,187,742 (75.4)
	1	426,677 (16.3)	27,329 (9.9)	454,006 (15.7)
	2	155,654 (5.9)	4647 (1.7)	160,301 (5.5)
	≥3	96,463 (3.7)	2054 (0.8)	98,517 (3.4)
**Education (years)**				
	<10	601,787 (22.9)	42,890 (15.6)	644,677 (22.2)
	10-15	1,175,364 (44.8)	124,310 (45.2)	1,299,674 (44.8)
	>15	807,635 (30.8)	105,160 (38.2)	912,795 (31.5)
	Unknown	40,577 (1.5)	2843 (1)	43,420 (1.5)
**Ethnicity**				
	Non-Western	195,638 (7.4)	21,790 (7.9)	217,428 (7.5)
	Western, not born in Denmark	54,761 (2.1)	5773 (2.1)	60,534 (2.1)
	Native, born in Denmark	2,325,363 (90.5)	247,640 (90)	2,622,604 (90.4)
**Income (quintiles)**				
	1	487,441 (18.6)	50,997 (18.5)	538,438 (18.6)
	2	598,552 (22.8)	45,088 (16.4)	643,640 (22.2)
	3	586,618 (22.3)	62,783 (22.8)	649,401 (22.4)
	4	540,238 (20.6)	65,139 (23.7)	605,377 (20.9)
	5	405,990 (15.5)	50,674 (18.4)	456,664 (15.7)
	Negative or zero	6524 (0.2)	522 (0.2)	7046 (0.2)
**Urbanization (population)**				
	>100,000	507,926 (19.4)	54,166 (19.7)	562,092 (19.4)
	20,000-100,000	655,472 (25)	67,516 (24.5)	722,988 (24.9)
	1,000-20,000	867,341 (33)	85,347 (31)	952,688 (32.9)
	<1,000	593,691 (22.6)	68,121 (24.8)	661,812 (22.8)
	Unplaceable	933 (0)	53 (0)	986 (0)
**Employment status**				
	Unemployed	332,428 (12.7)	27,305 (9.9)	359,733 (12.4)
	Retired	252,098 (20)	11,941 (4.3)	537,039 (18.5)
	Employed	1,767,837 (67.3)	235,957 (85.8)	2,003,794 (69.1)
**Time of contact (hours until opening time of daytime general practice)**				
	>4 before office hours	2,527,937 (96.3)	268,668 (97.6)	2,796,605 (96.4)
	<4 before office hours	97,426 (3.7)	6535 (2.4)	103,961 (3.6)
**Region**				
	North Denmark Region	405,869 (15.5)	34,556 (12.5)	440,415 (15.2)
	Central Denmark Region	989,549 (37.7)	88,821 (32.3)	1,078,370 (37.2)
	Region of Southern Denmark	946,931 (36.1)	134,029 (48.7)	1,080,960 (37.3)
	Region Zealand	283,014 (10.7)	17,807 (6.5)	300,821 (10.4)

### Proportion of Video Contacts

During the study period, 9.5% (275,203/2,900,566) of telephone triage contacts to OOH-PC were video contacts. After the introduction of video, a range of 5%-15% video contacts was achieved within weeks across all regions. This level remained stable throughout the study period (data not shown).

### Association Between Video Contact and Patient- and Contact-Related Characteristics

The frequency of video contacts was unevenly distributed across patient- and contact-related characteristics. The strongest associations were seen for age, comorbidity, employment status, region, and time of contact (Figure 1). Patients aged <20 years had a notably higher frequency of video contacts than patients aged 21 to 40 years (aIRR range: 2.39-1.31). This was also the case for employed compared to unemployed patients (aIRR 1.21). The frequency of video contacts was significantly higher for contacts to OOH-PC at more than 4 hours before the opening of daytime general practice (aIRR 1.40; reference: ≤4 hours), and was more frequent in the Region of Southern Denmark (aIRR 1.55; reference: North Denmark Region). In contrast, the frequency of video contacts was significantly lower for patients >40 years (aIRR range: 0.40-0.90; reference: 21-40 years), patients with comorbidities (aIRR range 0.59-0.90; reference: no comorbidities), and retired patients (aIRR 0.66; reference: unemployed). The frequency of video contacts was also significantly lower in Region Zealand (aIRR 0.73; reference: North Denmark Region).

### Triage Outcomes

Patients receiving a video contact had a significantly higher frequency of ending the contact with advice and self-care compared to patients receiving a telephone contact (aIRR 1.21, 95% CI 1.21-1.21) (Table 2). Conversely, patients receiving a video contact had a significant lower frequency of being referred to a clinic consultation (aIRR 0.59, 95% CI 0.59-0.60) or a home visit compared to patients receiving a telephone contact (aIRR 0.31, 95% CI 0.29-0.32). The frequency of being admitted to a hospital was significantly higher after a video contact compared to a telephone contact (aIRR 1.20, 95% CI 1.17-1.23).

**Table 2.** Frequency of triage outcomes and their association with video contacts (incidence rate ratio).

Outcome	Telephone contacts (n=2,625,363), n (%)	Video contacts (n=275,203), n (%)	Total (n=2,900,566), n (%)	Incidence rate ratio (95% CI)	
				Crude	Adjusted^a^
Advice and self-care	1,663,681 (63.4)	215,484 (78.3)	1,879,567 (64.8)	1.24 (1.23-1.24)	1.21 (1.21-1.21)
Clinic consultation	712,255 (27.1)	49,262 (17.9)	759,948 (26.2)	0.66 (0.66-0.67)	0.59 (0.59-0.60)
Home visit	165,052 (6.3)	1926 (0.7)	168,233 (5.8)	0.12 (0.11-0.12)	0.31 (0.29-0.32)
Hospital admission	84,375 (3.2)	8531 (3.1)	92,818 (3.2)	0.95 (0.93-0.97)	1.20 (1.17-1.23)

a Adjusted for patient sex, age, cohabitation status, comorbidity, educational level, ethnicity, income, urbanization, employment status, region, and time of contact.

### Follow-Up Contacts

In general, patients receiving a video contact had a significantly higher frequency of no follow-up contact compared to patients receiving a telephone contact (aIRR 1.09, 95% CI 1.08-1.09) (Table 3). For those who had a follow-up contact, the patients who received a video contact had a significantly higher frequency of having a follow-up contact with their regular GP compared to those receiving a telephone contact (aIRR 1.02, 95% CI 1.01-1.03). Conversely, patients receiving a video contact had a significant lower frequency of a follow-up contact in OOH-PC (aIRR 0.96, 95% CI 0.95-0.97) or at the hospital (aIRR 0.75, 95% CI 0.74-0.76) compared to patients receiving a telephone contact.

**Table 3.** Frequency of follow-up contacts and association between use of video contacts and subsequent follow-up contacts (incidence rate ratio).

Type of follow-up contact	Telephone contacts (n=2,625,363), n (%)	Video contacts(n=275,203), n (%)	Total (n=2,900,566)	Incidence rate ratio (95% CI)	
				Crude	Adjusted^a^
No follow-up	1,097,402 (41.8)	137,601 (50)	1,232,741 (42.5)	1.20 (1.19-1.20)	1.09 (1.08-1.09)
Daytime general practice^b^	719,349 (27.4)	70,728 (25.7)	791,854 (27.3)	0.94 (0.93-0.94)	1.02 (1.01-1.03)
OOH-PC^c^,^d^	396,430 (15.1)	37,703 (13.7)	435,085 (15)	0.90 (0.90-0.91)	0.96 (0.95-0.97)
Hospital^e^	412,182 (15.7)	29,171 (10.6)	440,886 (15.2)	0.68 (0.67-0.69)	0.75 (0.74-0.76)

a Adjusted for patient’s sex and age, cohabitation status, comorbidity, educational level, ethnicity, income, urbanization, employment status, region, and time of contact.

b Contacts (telephone contacts, video contacts, clinic consultations, or home visits) to daytime general practice within 7 days from the index contact to OOH-PC.

c OOH-PC: out-of-hours primary care.

d All telephone triage contacts to OOH-PC within 7 days from the index contact to OOH-PC.

e All nonscheduled hospital contacts (emergency department visits and hospital admissions) within 1 day from the index contact to OOH-PC.

## Discussion

### Principal Results

Video was used in 9.5% (275,203/2,900,566) of all telephone triage contacts to OOH-PC. Video contacts were unevenly distributed across patient- and contact-related characteristics; video contacts were more often used for patients who were employed, young, without comorbidities, and contacting OOH-PC more than 4 hours before the opening hours of daytime general practice. Compared to telephone contacts, significantly more video contacts ended with advice and self-care and significantly fewer had follow-up contacts.

### Strengths and Limitations

This study was based on a large data set, including codes for remuneration by GPs. The economic incentive for GPs to register all services provided contributed to the completeness of the data, though validity has not been studied [22].

Our study also had some limitations. First, we had no information on the reasons for encounters (RFEs), as this is not systematically registered in OOH-PC contacts. In each telephone triage contact, the triage GP assessed the relevance of video use based on the current RFE balanced against the specific patient- and contact-related characteristics. Therefore, telephone contacts and video contacts had different diagnostic scope, which could have influenced the differences found in triage outcome and follow-up contacts through confounding by indication. Second, we followed each patient for 7 days to record follow-up contacts to OOH-PC and to daytime general practice, as previously described in the literature [26]. This led to an overestimation of follow-up contacts, as we could not link these follow-up contacts to the index contact in OOH-PC using the RFE. However, any overestimation would be independent of type of contact. Finally, we used the Charlson Comorbidity Index to define comorbidity based on hospital diagnosis codes. This approach might have led to an underestimation of comorbidity [27], as patients with mild chronic diseases are often treated solely in general practice.

Several factors must be considered when generalizing the results of this study. First, the study period was defined according to the date of initiation of video contact in each of the regions. Therefore, the regions were included in different periods of the COVID-19 pandemic, and they had different contact patterns to primary care both inside and outside office hours [8,12,20,28] and probably also different distributions of triage outcomes. Second, triage GPs perform telephone triage with no decision support tool in Danish OOH-PC; this is unlike most countries with comparable OOH-PC services, which often use other health care professionals with decision support systems [3]. Compared to other triage professionals, GPs may be able to triage more patients via video contact. Lastly, Danish triage GPs were remunerated on a fee-for-service basis. As the fee for a video contact was higher than the fee for a telephone contact, this could have been an incentive to aim for a higher share of video contacts in this setting compared to countries with other payment structures.

### Comparison With Prior Work

We found a 9.5% rate of use of video contacts to OOH-PC. To our knowledge, no previous studies used a data collection period of this length to report on video use in OOH-PC. Studies on changing contact patterns in OOH-PC during the COVID-19 pandemic have found an overall increase in telehealth consultations (email, video, or telephone) [8,12,28]. Video use in daytime general practice has previously been reported to range from 1% to 6.4% [15,29-32]. However, as patient populations and RFEs are known to differ between daytime general practice and OOH-PC [33], these results cannot be compared with our findings. Furthermore, video contacts in OOH-PC guide triage professionals in the assessment of patients and in improving patient reassurance [13,14]. In contrast, video contact has often been used as a substitute for clinic consultations in daytime general practice for practical reasons, for example, to reduce travel time or limit the risk of contamination, but both patients and GPs seem to prefer in-person consultations in the postpandemic era [10,13,18].

Our study showed that video contacts were used more often for employed young patients without comorbidities. To the best of our knowledge, this is the first study to report on associations between patient- and contact-related characteristics and video contacts in OOH-PC. Studies conducted in daytime general practice have found higher video rates of use during COVID-19 lockdown periods [34] and among people from socioeconomically advantaged areas [34,35]. Previous studies have reported inconsistent results on the association between patient age and video use, as higher use has been reported for both younger [32,35] and older patients [30]. Moreover, daytime video use seems to be associated with patients with high morbidity [36] compared to patients with low morbidity. These findings are not in line with our study results, which could be due to differences in patient populations between daytime general practice and OOH-PC [33]. Furthermore, some previous studies were conducted during the peak of the COVID-19 pandemic, and different countries have different health care systems and had different approaches to tackling the pandemic.

We found that video contacts more often ended with advice and self-care and no follow-up contact compared to telephone contacts. Two qualitative studies investigating the effect of video contacts on the patient flow in daytime general practice found that GPs experience uncertainties when referring patients to secondary care after a video contact [9,37]. A UK study on follow-up contact after using a video contact service (used by hospitals, daytime general practices, and other services) found no significant difference in the number of subsequent referrals compared to telephone contacts [38]. However, these studies focused on video use in the daytime rather than on telephone triage in OOH-PC.

### Implications for Practice and Future Research

Our study suggests that video contacts could help reduce the use of health care resources in the OOH-PC setting by lowering the number of subsequent clinic consultations and home visits. More studies are needed on the effect of video contact on patient flow. First, further research is needed to investigate the impact of video contact in relation to different RFEs. Second, future studies should explore if the findings of this study are maintained in the postpandemic period and across different OOH-PC organizations. Third, future studies should investigate if the video option might generate more contacts to OOH-PC overall. Fourth, our study indicates an association between video contacts and specific patient characteristics: video was more often used for employed young patients without comorbidities. This finding contrasts with most studies in daytime general practice and should be further investigated. Finally, it is important to note that we did not study costs associated with video use and its effects on resource use. Therefore, future studies should investigate the costs as well.

### Conclusion

This study supports our hypothesis that video contacts could reduce use of health care resources in OOH-PC. Video use lowered the frequency of referrals to a clinic consultation or home visit and also lowered the frequency of follow-up contacts. However, the results could be biased due to confounding by indication, reflecting that triage GPs use video for a specific set of RFEs.

## Abbreviations

aIRR: adjusted incidence rate ratio
GDPR: General Data Protection Regulation
GP: general practitioner
ICPC-2: International Classification of Primary Care, 2nd Edition
IRR: incidence rate ratio
OOH-PC: out-of-hours primary care
RFE: reason for encounter
STROBE: Strengthening the Reporting of Observational Studies in Epidemiology
